# The impact of cultivation systems on the nutritional and phytochemical content, and microbiological contamination of highbush blueberry

**DOI:** 10.1038/s41598-020-73947-8

**Published:** 2020-10-07

**Authors:** Ireneusz Ochmian, Magdalena Błaszak, Sabina Lachowicz, Renata Piwowarczyk

**Affiliations:** 1 Department of Horticulture, West Pomeranian University of Technology Szczecin, Słowackiego 17 Street, 71434 Szczecin, Poland; 2 Department of Bioengineering, West Pomeranian University of Technology Szczecin, Słowackiego 17 Street, 71434 Szczecin, Poland; 3 Department of Fermentation and Cereals Technology, Wrocław University of Environmental and Life Sciences, Chełmońskiego 37 Street, 51630 Wrocław, Poland; 4 Institute of Biology, Jan Kochanowski University, Uniwersytecka 7 Street, 25406 Kielce, Poland

**Keywords:** Secondary metabolism, Agroecology

## Abstract

The aim of this study was to compare the nutritional and use value of berries grown in organic and conventional systems. The polyphenol content, fruit colour and firmness, and yeast, mould, and mycotoxin contents were assessed in blueberry fruit freshly harvested and stored for 8 weeks in controlled conditions (CA: CO_2_-12%; O_2_-1.5%, temperature 1.5 °C). The Shannon–Wiener diversity index was comparable in both systems and was lower for mould in organic fruit than in conventional fruit. Mycotoxins (deoxynivalenol, zearalenone) were found only in organic fruits. The optimal mineral content and pH of the soil allowed the cultivation of blueberry in accordance with organic standards. The storage of highbush blueberry fruit in CA cold storage for 8 weeks resulted in a slight deterioration in fruit quality and polyphenol content. The lower nutritional value of these fruits is compensated by the lack of pesticides and artificial fertilizers residues. The use of fungicides in conventional cultivation reduces the population of sensitive fungi and therefore reduces the contamination of fruits with mycotoxins.

## Introduction

Global production of the northern highbush blueberry (*Vaccinium corymbosum* L.) approaches nearly 655 thousand tons per year, and it has increased 20-fold within the last 20 years. Highbush blueberry production and processing occurs primarily in North America^1^. The area under cultivation has increased substantially in China, Mexico, Poland and Spain^1^. Growing demand for blueberry stems from the unique nature of the fruit, which has no commercial equivalent, its considerable nutritional value and high content of polyphenols, especially anthocyanins^2^.

The demand for organic fruits is growing every year. This is due to the raising awareness of consumers who see the logical link between polluted agricultural produce, air and water, the increasing incidence of cancers, allergies and infertility^3,4^. When optimum conditions are created for the growth and development of plants, organically grown fruit can attain very high quality, higher than that in conventional cultures, which is contrary to the conventional wisdom^5^. Globally, approximately 5% of the total production area of blueberry is organically cultivated. Blueberry cultivation, also ecological, is not particularly demanding. First and foremost, an optimum soil (humus-rich and acidic) should be prepared for proper growth^6^. Problems in the organic cultivation of the fruit arise especially during humid and warm years, with perfect conditions for mould fungi to colonize on the aboveground parts and the roots. Additionally, fungi as well as blueberry, prefer slightly acidic substrate/soil^7^.

The weather is an important factor that has a direct impact on cultivated plants in recent years, it has become increasingly unpredictable. The increase in temperature is not the only issue. The considerable variations in precipitation levels during fruit ripening in individual years also pose problems^8^. Blueberry cultures face particular danger from *Glomerella acutata* (*anthracnose*),* Botrytis cinerea* (gray mould), *Godronia cassandrae* (godronia canker), *Botryosphaeria corticis* (canker), *Phytophthora* spp. (root rot), *Diaporthe* spp. (twig blight and fruit rot), and *Alternaria* *tenuissima*^9^. In integrated organic cultivation, monitoring is the basis for the estimation of pest/phytopathogen infestation in the crop, as well as for the analysis of plant yield to determine potential causes for yield loss^10^. The fungal infection and the growth of mould on the plant results in financial losses for the farming industry and poses a threat to consumer’s health. To become a source of mycotoxins, the mycelium does not have to be highly developed and thus visible to the consumer. The fungus grows into the substrate/product and penetrates the tissues and even the cells of the plant. The substrate mycelium is responsible for nurturing the entire mycelium, as well as the surface mycelium^4^. Research shows that fruit, even when stored under refrigerated conditions, maybe a source of hazardous mycotoxins^11,12^. Fungal infection of the blueberry fruit may be visible and/or organoleptically detected (visible fungal mycelium, changes in fruit and juice colour due to flavonoid decomposition)^13–15^. Worse still, fungal infection also may remain invisible, posing a health hazard. Mycotoxins have deleterious effects on human internal organs; they are invisible and not sensorically perceptible^4,16^.

The aim of this study was to compare the nutritional and use value of berries grown in organic and conventional systems. A wide spectrum of the polyphenol fraction was selected as the comparison parameter, and the antioxidant activity and anti-diabetic effects of fresh highbush blueberry fruit were evaluated at harvest and after of controlled atmosphere (CA) storage. We also evaluated whether contact with fungicides reduced the mycotoxin content by restricting mould development.

## Results

### Уeasts, moulds and mycotoxins

The number of yeasts isolated from fruit varied widely (2.7–4.8 log_10_ CFU/g) (Table 1). Generally, it can be said that the count of yeasts isolated from organic fruit was lower than that isolated from conventionally grown fruit (both fresh and stored). On average, 0.5 log_10_ CFU/g less yeast inhabited fresh organic fruit than conventionally cultivated fruit (the latter fruits contained an average of 4.0 log_10_ CFU/g of yeast). After a period of cold storage, the number of yeast on fruit significantly decreased in most cases (Table 1). For example, on fresh organic fruit from field No. 1, the count of yeast was 3.1 log_10_ CFU/g; after storage, the number dropped by 0.4 log_10_ CFU/g. A similar decrease in the amount of yeast after storage was recorded on fruits from the second organic and second conventional fields. In the case of fruit from conventional field No. 1, the number of yeasts significantly increased by 0.5 log_10_ CFU/g with the storage time of these fruits. The amount of mould grown on blueberry fruit, like the number of yeasts, was characterized by a wide range (1.8–3.9 log_10_ CFU/g) (Table 1). Even fruits taken for analysis from the same field at different points (1 or 2) differed significantly in their mould content, and these differences were statistically significant. The largest difference was 1.9 log_10_ CFU/g between fresh organic fruit harvested from fields No. 1 and No. 2. As with yeast, more mould was found on the conventionally cultivated fruit (both freshly harvested and stored) than on the organically cultivated fruit. After the cold storage period, the amount of mould on the fruit did not change or significantly decrease (Table 1). The largest decrease in the amount of mould after storage was observed on organic fruits harvested from field No. 2—by 1.3 log_10_ CFU/g (Table 1). Fungi of the genus *Cladosporium* were most frequently found in organically grown fruit (45–84% of all identified fungi). In conventionally grown fruit, *Aureobasidium* was the most commonly found fungus (Table 1). Fungi belonging to eight different genera (*Cladosporium, Fusarium, Penicillium, Acremonium, Alternaria, Aureobasidium, Bipolaris,* and *Mucor*) were isolated from organic fruits, and five genera of fungi were isolated from conventionally cultivated fruits (*Cladosporium, Penicillium, Alternaria, Aureobasidium, Eurotium*). Although more types of fungi were found on the organic fruit, the vast majority belonged to one dominant genus, i.e., *Cladosporium* (Table 1). This is why the mould from organic fruit had a low value for the biodiversity index (in the case of organic fruits from field No. 2, the Shannon–Wiener index = 0.284 ± 0.095). Conventionally cultivated fruits were also inhabited by large quantities of fungi belonging to the genus *Aureobasidium*; therefore, a higher Shannon–Wiener diversity index value was obtained. After storage, the moulds found on the fruit belonged to only 2–3 genera (in the case of conventionally cultivated fruit, 88–99% of isolates belonged to *Aureobasidium*). *Fusarium* was not present in the conventionally cultivated fruit, and this genus could not be identified in fresh or stored fruit, or in stems or leaves. Interestingly, *Fusarium* fungi were isolated from fruits on one side of the organic field. The total count of moulds on these fruits did not decrease even after 8 weeks of cold storage, and the share of *Fusarium* in the total number of fungi colonizing the fruit increased from 15 to 27%. The presence of deoxynivalenol, a *Fusarium* mycotoxin, was also identified in these samples (mean 2.59 ± 0.14 and 1.59 ± 0.13 µg/kg in fresh and stored fruit). Zearalenone, another toxin, was detected only after the storage period and was not found in fresh fruit. No ochratoxin A, toxin T2 HT2, aflatoxin (BI, B2, GI, G2), or patulin were identified in the tested samples of fresh and stored fruit.

**Table 1:** Impact of the cultivation method and storage of blueberry fruit on the amount of yeast and mould and the composition and variety of fungi as well as the presence of mycotoxins in fruit.

Blueberry fruit	Уeast log_10_CFU/g		Mould log_10_CFU/g	Fungi isolated from fruit (affiliation to genus, % of all isolated)			Shannon–Wiener index	Mycotoxins µg/kg
**Organic cultivation**								
Fresh	1^a^	3.1e^b^	1.8d	63	*Cladosporium*	0.457 ± 0.037		Deoxynivalenol 2.59 ± 0.14^c^
				15	*Fusarium*			
				13	*Penicillium*			
				9	*Acremonium*			
	2	3.9c	3.7a	84	*Cladosporium*	0.284 ± 0.095		–
				6	*Penicillium*			
				5	*Alternaria*			
				2	*Bipolaris*			
				2	*Acremonium*			
				1	*Mucor*			
Stored	1	2.7f	1.7d	45	*Cladosporium*	0.458 ± 0.024		Deoxynivalenol 1.59 ± 0.13 Zearalenone 0.16 ± 0.01
				28	*Aureobasidium*			
				27	*Fusarium*			
	2	3.4de	2.4cd	76	*Cladosporium*	0.237 ± 0.036		–
				24	*Aureobasidium*			
**Conventional cultivation**								
Fresh	1	3.2e	3.3ab	45	*Aureobasidium*	0.476 ± 0.060		–
				37	*Cladosporium*			
				15	*Alternaria*			
				3	*Penicillium*			
	2	4.8a	3.9a	45	*Cladosporium*	0.462 ± 0.047		–
				42	*Aureobasidium*			
				8	*Penicillium*			
				5	*Eurotium*			
Stored	1	3.7d	2.6cd	88	*Aureobasidium*	0.152 ± 0.075		–
				12	*Alternaria*			
	2	4.5b	3.9d	99	*Aureobasidium*	0.024 ± 0.001		–
				1	*Cladosporium*			

^a^1–2 field number.

^b^Values followed by the same letter, within the same column, were not significantly different (p < 0.05) according to t-Tukey test.

^c^Mean values ± SD.

Fungi of the genus *Aureobasidium* commonly colonized the fruit, and their share of the total fungi increased considerably in the stored fruit originating from both organic and conventional cultures. In fresh organically cultivated fruit, they constituted less than 1% of all isolated strains, whereas in the same fruit after 8 weeks of storage, they increased to over 20% of the total fungi. Side shoots with leaves and main shoots of organically grown blueberries were inhabited by a more diverse group of moulds than those of conventionally grown blueberries. Moulds of the genus *Fusarium* sp., *Triposporium* sp. *Pestalotiopsis maculans*, *Stemphylium* sp., were only detected in plant material from the organic farm (Table 2).

**Table 2:** Differences in the mould composition from the leaves and shoots of the highbush blueberry, depending on the method of cultivation.

Part of the plant	Dominant fungal species	
	Organic cultivation	Conventional cultivation
Side shoots with leaves	*Alternaria alternata* *Botrytis cinerea* *Fusarium* sp. *Cladosporium herbarium*	*Alternaria alternata* *Botrytis cinerea*
Main shoots	*Phomopsis vaccinni* *Phytophtora* sp. *Rhizoctonia solani* *Triposporium* sp. *Pestalotiopsis maculans* *Stemphylium* sp.	*Phomopsis vaccinni* *Phytophtora* sp. *Rhizoctonia solani* *Colletotrichum gleosporioides* *Botrytis cinerea*

### Firmness, color, and weight loss of fruit

**Figure 1:**
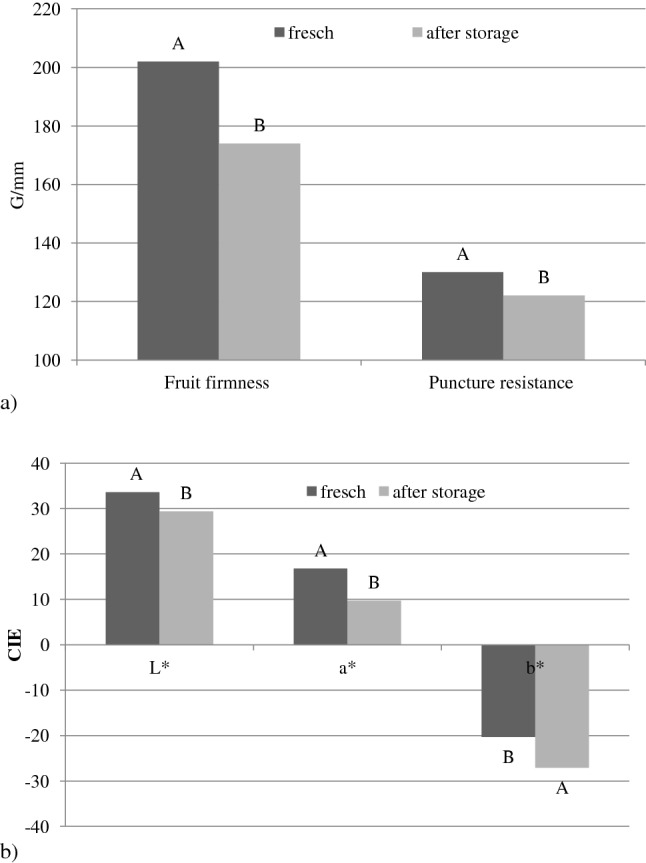
Changes in physical (**a**) and colour parameters (**b**) of highbush blueberry fruit after CA cold storage.

The influence of the cultivation method and storage time on the physical parameters of the fruit was also examined (Tables 3 and 4). It is difficult to determine whether the confirmed presence of moulds, yeasts, and yeast-like fungi in the examined fruit affected their tenderness or colour. After 8 weeks of CA cold storage, firmness decreased by less than 14% (Fig. 1). Fruit size has a decisive impact on fruit quality. Smaller fruits harvested on a conventional plantation were firmer and less prone to mechanical damage (Table 3), and had higher amounts of bioactive compounds. These fruits remained firm after 8 weeks of storage (Table 4). Despite the variation in the levels of specific pathogens on fruits from different fields, their firmness was similar, as was their resistance to mechanical damage. However, organically-grown fruits from different fields (1 and 2) differed in firmness. Fruits from field 2 were less firm and had a greater number and diversity of fruit-colonizing fungi. However, these differences became insignificant after storage. Changes in fruit firmness can be caused by many factors, which may result in the deterioration of fruit quality. This may also have resulted in slightly higher weight loss after storage and a greater diversity of yeasts and moulds. The fruit weight losses in the tested cultivar were relatively low, with means in the range of 1.4–2%. The greatest weight loss was found in fruits harvested from the first plot of the organic plantation (2.4%). A higher biodiversity of fungi and their metabolites (mycotoxins, deoxynivalenol and zearalenone) were found on these fruits.

**Table 3:** Quality and colour of fresh blueberry fruit, depending on the cultivation method.

Parameters	Fields number	Organic cultivation	Conventional cultivation	Mean
Weight of 100 berries (g)	1	365a^a^	227b	296A
	2	388a	196b	292A
	Mean	377A	212B	
Fruit firmness (G/mm)	1	189b	217a	203A
	2	172c	230a	201A
	Mean	180B	224A	
Puncture resistance (G/mm)	1	137a	125a	131A
	2	126a	131a	128A
	Mean	132A	128A	
**Color fruits parameters**				
L* white 100 black 0	1	32.9bc	34.8ab	33.9A
	2	31.5c	35.2a	33.4A
	Mean	32.2B	35.0A	
a* green − 100 red + 100	1	16.5a	17.7a	17.1A
	2	17.9a	15.2a	16.6A
	Mean	17.2A	16.5A	
b* blue − 100 yellow + 100	1	− 18.2c	− 22.4a	− 20.3A
	2	− 20.5ab	− 19.9bc	− 20.2A
	Mean	− 19.4A	− 21.2A	

^a^Mean values denoted by the same letter do not differ statistically significantly at 0.05 according to t-Tukey test; lower-case letters indicate interaction and capital letters the main factors.

**Table 4:** Differences in the quality and colour of highbush blueberry fruit after CA cold storage, depending on the cultivation method.

Parameters	Fields number	Organic cultivation	Conventional cultivation	Mean
Fruit firmness (G/mm)	1	156b^a^	192a	174A
	2	141b	205a	173A
	Mean	149B	199A	
Puncture resistance (G/mm)	1	122a	119a	121A
	2	118a	127a	123A
	Mean	120A	123A	
Weight losses (%)	1	2.4c	1.3a	1.8A
	2	1.7b	1.5ab	1.6A
	Mean	2.0A	1.4B	
**Color fruits parameters**				
L*white 100 black 0	1	24.7b	31.7a	28.2B
	2	27.6b	33.4a	30.5A
	Mean	26.2A	32.6B	
a*green − 100 red + 100	1	7.4c	11.3a	9.4A
	2	9.1b	10.8b	10.0A
	Mean	8.3B	11.1A	
b*blue − 100 yellow + 100	1	− 29.3a	− 27.2b	− 28.3A
	2	− 26.7bc	− 25.0c	− 25.9B
	Mean	− 28.0A	− 26.1A	

^a^For explanation, see Table 3.

Independent of the cultivation method, the fresh fruits had similar L*a*b* colour parameters. Though similar in colour, the fruits harvested from conventional plantation had considerably higher contents of polyphenol compounds, including anthocyanins. The difference was significant, with 313.71 mg/100 g of anthocyanins determined in the organic fruit and 448.65 mg/100 g determined in the conventional fruit. Anthocyanins are largely responsible for fruit colour. The fruits, especially the organic fruits, darkened during storage (Fig. 1).

A change in the L* colour parameter indicates that the blueberry fruit is darkening. Irrespective of the cultivation method, the fruits changed from reddish to blue shades, as indicated by considerable changes in the a* and b* colour parameters. These changes were more pronounced in organic fruit than in conventional fruit. However, the changes in the content of anthocyanins were more pronounced in the conventional fruit, where the anthocyanin content decreased by 44% compared to that in fresh fruit.

The study demonstrated that storage had little effect on the reduction of the antioxidative effects of the blueberry fruits as determined by ABTS·+, DPPH and FRAP (Table 5). A decrease in inhibitory activities was observed after the storage period, but mainly in the organic fruits (*α*-amylase IC_50_ 27.33, *α*-glucosidase IC_50_ 19.68 mg/mL). In conventional fruits, the level of inhibitory activities was higher, at 20.63 and 15.07 mg/mL, respectively. The changes in antioxidative effects and inhibitory activities may have resulted from the decrease in polyphenol content as well as from the decrease in *L-*ascorbic acid in fruit after the storage period. Contrary to popular opinion, the fruit from the conventional plantations had higher nutritional value, i.e., higher antioxidant activity, anti-diabetic effects and polyphenol content (Tables 5 and 6).

**Table 5:** Changes in health promoting capacities of highbush blueberry fruit after CA cold storage.

	Organic cultivation		Conventional cultivation	
	Fresh fruits	Stored fruits	Fresh fruits	Stored fruits
ABTS·+ (μmol/g)	19.33b^a^	17.06c	22.57a	21.88a
DPPH (μmol/g)	17.42b	15.69b	20.44a	19.83a
FRAP (μmol/g)	7.61b	7.94b	9.11a	9.35a
*α*-amylase IC_50_ (mg/mL)	24.48b	27.33a	22.28bc	20.63c
*α*-glucosidase IC_50_ (mg/mL)	18.47b	19.68a	15.53c	15.07c
*L-*ascorbic acid (mg/100 g)	128c	115d	168a	142b
NO_3_ (mg/1000 g)	43.1b	48.9a	35.5c	40.7b
NO_2_ (mg/1000 g)	0.17c	0.19bc	0.21b	0.24a

^a^Values followed by the same letter, within the same line, were not significantly different (p < 0.05) according to t-Tukey test.

**Table 6:** Polyphenol content in fresh and stored blueberry fruit cultivated organically and conventionally (mg/100 g).

	Organic cultivation		Conventional cultivation	
	Fresh fruit	Stored fruits	Fresh fruit	Stored fruits
Caffeoyl-glucose	1.46a^a^	0.47c	0.70b	0.28d
Caffeoyl-glucose	3.92c	4.88b	8.57a	3.96c
Caffeoyl-glucose	0.51d	0.94c	2.01a	1.75b
Neochlorogenic acid	5.20a	2.29c	3.04b	3.32b
Chlorogenic acid	232.41a	181.32c	230.41a	217.27b
Cryptochlorogenic acid	1.40b	0.53d	1.17c	2.62a
Sum of phenolic acids	244.90A	190.43B	245.90A	229.20A
Myricetin 3-*O*-galactoside	3.24c	2.86d	10.19a	8.54b
Quercetin diglucoside	0.99a	0.34d	0.63c	0.82b
Quercetin 3-*O*-rhamno-hexoside	5.36a	3.98b	1.47c	0.99d
Quercetin 3-*O*-rutinoside	2.94a	2.69abc	2.25b	0.60c
Quercetin 3-*O*-galactoside	3.95d	9.10c	16.21a	11.52b
Quercetin 3-*O*-methoxyhexoside	2.57c	5.08a	3.70b	3.52b
Quercetin 3-*O*-glucoside	0.88c	1.60a	1.26b	0.62ad
Quercetin 3-*O*-arabinoside	3.51b	5.92a	3.08c	2.03d
Quercetin 3-*O*-caffeoylgalactoside	1.02a	0.91b	0.57c	0.27d
Quercetin 3-*O*-caffeoylglucoside	0.58a	0.46b	0.27c	0.21c
Quercetin 3-*O*-oxalylpentoside	5.28b	13.17a	3.05c	0.11d
Quercetin 3-*O*-rhamnoside	0.47c	1.05b	0.52c	1.87a
Quercetin 3-*O*-dimethoxyrhamnoside	0.89b	3.44a	0.06d	0.54c
Quercetin 3-*O*-(6′-acetyl)galactoside	0.07c	0.47a	0.12b	0.13b
Quercetin 3-*O*-(6′-acetyl)galactoside	0.34b	0.53a	0.03c	0.02c
Sum of flavonols	32.09C	51.60A	43.41B	31.79C
Procyanidin B dimer	4.99b	6.46a	5.15b	5.29b
Procyanidin B dimer	4.56b	5.71a	5.65a	3.92c
Procyanidin B dimer	11.16b	57.80a	13.70c	26.07b
(+)-Catechin	11.12b	5.64c	3.41d	27.35a
(−)-Epicatechin	3.63c	9.95a	9.54a	5.45b
Procyanidin trimer—Procyanidin B3	0.76c	3.74a	3.84a	2.59b
Sum of flavan-3-ols	36.22C	89.3A	41.29C	70.67B
Delphinidin-3-*O*-glucoside	84.32b	46.8c	109.16a	51.04c
Delphinidin 3-*O*-arabinoside	1.97a	1.68b	2.08a	2.04a
Petunidin-3-*O*-glucoside	59.23b	33.43d	82.06a	43.51c
Cyanidin-3-*O*-glucoside	41.45b	35.09c	58.49a	33.07c
Cyanidin 3-*O-*arabinoside	7.34c	13.73b	13.72b	23.25a
Petunidin 3-*O-*arabinoside	23.96b	20.08b	37.22a	23.30b
Malvidin 3-*O-*galactoside	72.72b	42.77d	89.68a	58.63c
Malvidin 3-*O-*arabinoside	4.14d	4.72c	5.99b	7.44a
Malvidin-3-*O*-glucoside	28.58c	18.49d	50.25a	35.33b
Sum of anthocyanins	313.71B	216.79C	448.65A	275.97B
Total	636.92B	548.12C	779.25A	607.63B

^a^For explanation, see Table 5.

### Polyphenol compounds and health promoting capacities

Fruits harvested from the test fields of the organic as well as the conventional plantation (fields 1 and 2) were characterized by similar polyphenol compound contents (Table 6). The profile of polyphenols in blueberry fruits included six phenolic acids, including hydroxycinnamic acid, and their derivatives (three compounds), flavonols and their derivatives (15 compounds), flavan-3-ols (six compounds), and anthocyanins (nine compounds) (“Supplementary Information”). The polyphenol profiles of the organic and conventional fruit were identical. However, the total polyphenols were 22% higher in fruits harvested from conventionally grown bushes. During CA cold storage, the amount of polyphenols was reduced. Regardless of the cultivation method, anthocyanins comprised the largest group of the identified compounds. Anthocyanins also showed considerable changes in amount after storage. In fresh organic fruit, the anthocyanin content was 313.71 mg/100 g, and in conventional fruit, it was 448.65 mg. After 8 weeks of CA cold storage, the content of anthocyanins decreased by 31% and 38%, respectively. The amount of delphinidin-3*-O*-glucoside decreased in both organic and conventional fruits. Moreover, considerable quantitative and percentage changes occurred in the contents of petunidin-3*-O*-glucoside and malvidin 3-galactoside. Blueberry fruits were also rich in phenolic acids, especially chlorogenic acid (181.32–232.41 mg/100 g), which is classified as a subclass of hydroxycinnamic acids. Together with anthocyanins, phenolic acids made up 74–89% of all the identified polyphenols. The phenolic acid content was similar in both types of fresh fruit (Table 6). However, the amount of the following phenolic acids decreased in organic fruit after storage: neochlorogenic, chlorogenic, and cryptochlorogenic acids. On the other hand, the content of cryptochlorogenic acid increased in conventional fruit, from 1.17 at harvest to 2.62 mg/100 g after storage. Similar trends were observed for flavan-3-ols. After storage, the procyanidin content (T.r. 3.96) increased considerably in organic and conventional fruits, from 11.16 to 57.80 mg/100 g in organic fruit and from 13.70 to 26.07 mg/100 g in conventional fruit. Moreover, the catechin content was found to increase considerably in conventional fruit after storage.

Conventionally grown highbush blueberry fruits were characterized by higher antioxidant activity than organically grown fruits as determined using ABTS·+, DPPH and FRAP tests. However, this may have resulted from the fact that the organic fruits were much larger (mean weight of 100 fruits 377 g) than the conventional fruits (212 g). In the FRAP assay, the ability of blueberry extracts to reduce Fe3^+^ to Fe2^+^ ranged from 7.61 to 9.35 μmol/g. The free radical scavenging activity determined by DPPH varied from 15.69 to 20.44, and the values determined by ABTS·+ ranged from 17.06 to 22.57 μmol/g (Table 5). Storing conventionally grown fruit in CA cold storage did not have a great impact on the polyphenol and *L-*ascorbic acid content, antioxidant activity or anti-diabetic effects of the fruit. A slight decrease in the *α*-amylase and *α*-glucosidase inhibitory effects was found in the organic fruits. *L-*ascorbic acid and polyphenols (especially anthocyanins) are characterized by high antioxidant activity. The conventional fruit was characterized by a higher content of these compounds, potentially resulting in higher antioxidant activity. Polyphenols, especially anthocyanins, can affect antioxidant activity. Anthocyanins are mostly found in the skin of berries. The high amount of phenolic compounds can have a positive effect on health. Berries from organic plantations were larger than those grown on conventional farms. To grow blueberries organically, the right conditions must be met. The most important factors are a suitable humus content in the soil, low soil pH and the location of the plantation. Bushes on the organic plantation had these optimal conditions—the substrate had low pH and high humus content. This resulted in the production of large fruit. The bushes on the conventional plantation grew in sandy soil, which had to be supplied with water and fertilizer.

It should also be noted that the fruits contained very low levels of harmful nitrates and nitrites. In fresh fruit, the organically grown fruit had the highest level of nitrates (43.1 mg/1000 g), while conventional fruits had higher levels of nitrites (0.21 mg/1000 g). There was a slight increase in these substances during storage, but their levels remained significantly below the standards.

## Discussion

### Уeasts, moulds and mycotoxins

Typically, saprophytic genera (*Penicillium, Alternaria, Acremonium, Mucor, Aureobasidium*), and phytopathogenic fungi (*Bipolaris* sp*., Botrytis cinera, Phomopsis vaccinni, Phytophtora* sp*., Rhizoctonia solani, Cladosporium* sp*., Fusarium* sp.) were isolated from fruits, side shoots with leaves, and main shoots^10,17^. These fungi are commonly found on fruit, in soil and on agricultural products and plant materials regardless of the climatic zone^13,18,19^. Fresh fruits, including blueberries, demonstrate considerable tendency to infection by fungi that occurs during cultivation, harvest, transport, sale, and preparation for consumption. Visual and compositional fruit quality, as well as nutritive value changes, normally take place during storage.

Fruits have many simple sugars and organic acids; therefore, they are a good medium for yeast and mould. Colonization of the blueberry fruit by fungi depends on microhabitat conditions, numerous abiotic and biotic factors, and their interactions^13^. Ecological agriculture systems that do not include the use of pesticides and synthetic fertilizers are thought to promote the biodiversity of a given habitat^20,21^. The dominant type of fungi in organic cultivation was *Cladosporium* sp. (63–84% of all isolated fungi), and the fruit in conventional cultivation was most frequently inhabited by fungi from two genera: *Cladosporium* sp. (37–45%) and *Aureobasidium* sp. (42–45%). Other fungi belonging to other genera constituted only over a dozen percent of all isolated individuals. Because there was one clearly dominant genus—*Cladosporium* sp.—the biodiversity of organic fruit fungi (measured by the Shannon–Wiener diversity index) was comparable to or even lower than that of conventional fruit. The Shannon diversity index is high when there is a significant number of different species with individuals of similar abundance^20^. Therefore, our observation does not support the idea that the ecological system of plant cultivation supports biodiversity^21^. However, fungi belonging to 8 different genera were isolated from organic fruit, while only five genera were isolated from conventional fruit. Similarly, a richer set of taxa characterized the fungi isolated from organic plants (side shoots with leaves and main shoots). In addition, it should be noted that certain fungi (*Bipolaris*,* Acremonium*,* Mucor*,* Fusarium*) were found only on organic fruit. Considering the above, along with the observations and results of other authors^22^ it can be assumed that the cultivation system (organic or conventional) had a significant impact on fungi. The cultivation system significantly affected the number of yeasts and moulds as well as the taxonomic composition of fungi. However, this impact could not always be considered as promoting biodiversity, as evidenced by the Shannon–Wiener diversity index.

The strong competition among these fungi does not allow the excessive development of a selected group of phytopathogens^17,23^. However, the presence of mycotoxins has been detected in ‘Brigitta Blue’ fruit grown organically. This was probably related to the presence of fungi of the genus *Fusarium* on these fruits^24^. These fungi were only present on fruits from the organic farm; the fungicides used in conventionally grown fruit could have eliminated *Fusarium*. Therefore, it is very important to test fruit for the presence of mycotoxins, especially fruits from organic farms, before they reach consumers. Both types of fruit may pose a threat to the consumer: fruits from conventional crops may have pesticide residues, and fruits from organic farms may contain mycotoxins^12,25^. A well-known thesis was confirmed that the use of pesticides and synthetic fertilizers (strong interference with natural plant growth) changes the plant microbiome^22,23^. The same cultivar of blueberries, cultivated on the same soil, but in a different way, differed in the composition of fungi. Fewer types of fungi were found on chemically protected fruits and green shoots of blueberries than on organic ones. This was due to the sensitivity of fungi to pesticides^23^. However, greater share of various fungi on organic fruits had a certain negative effect—the appearance of toxin-forming *Fusarium* fungi, and consequently mycotoxins. This is a serious problem for organic farming. It turns out that not always natural mechanisms of competition between organisms, that should not allow excessive development of pests and phytopathogens, have a positive effect^17,23^. In our organic cultivation, this mechanism also did not work, and no share of different types fungi in the general composition was observed (Shannon–Wiener index). Only *Cladosporium* dominated on fresh organic fruit. This observation emphasizes the need for analytical tests for the presence of mycotoxins before organic fruit reaches the consumer.

Even 8 weeks of fruit storage (1.5 °C ± 0.25 °C) did not eliminate *Fusarium*, and consequently deoxynivalenol (1.59 ± 0.13 µg/kg) and zearalenone (0.16 ± 0.01 µg/kg) were found in the stored fruit. Refrigeration conditions do not eliminate toxin-producing fungi^13,26^.

Mycotoxins were found in the organic fruit; however, they were present at a low level. One reason for the low contamination level of 'Brigitta Blue' fruit with secondary mould metabolites might be the predominance of yeasts and yeast-like fungi from the genus *Aureobasidium*. They can degrade mycotoxins through a microbiological pathway^27,28^. Fungi inhabited both fresh fruit and those stored in CA cold storage. They were found in fruits from organic and conventional plantations. Some of them (*Aureobasidium*) showed a significant increase in abundance after 8 weeks of storage. Chi et al.^29^ noted that *Aureobasidium* fungi, including *A. pullulans*, exhibit a very strong antagonism to fruit-contaminating moulds and, therefore, may provide effective biological protection. However, the current study does not unambiguously confirm this thesis. *Aureobasidium* was the predominant fungal taxon under refrigerated conditions, occupying the niches left by other fungi that were sensitive to the cold. However, it turned out that the fungi of the *Aureobasidium* genus were not antagonists of *Cladosporium*; as in the majority of cases, they jointly colonized the fruit and dominated the other fungal taxa. Moreover, *Aureobasidium* did not inhibit the development of *Fusarium*: in the organic fruits evaluated after cold storage, each of these fungi made up 27% of the total fungal community, and the mycotoxins produced by *Fusarium* were also present.

### Fruit firmness, colour and weight loss

Blueberry fruit contaminated with fungi may lose some nutritional value and firmness due to the enzymatic activity of fungi^13^. Mould fungi penetrate deep into plant tissues through microinjuries and secrete enzymes, such as pectinases, lipases, and cutinases, to damage the epidermis of the berries^30^. Loss of firmness may also occur due to fruit decomposition and metabolite secretion^31^. Due to blueberry water loss sensitivity, long storage can develop wilting and softening symptoms which has a negative effect on firmness. Postharvest water loss and turgor pressure drop are more dramatic for the harvested crops compared with water stress occurring in the field, because of the cell’s inability to replace the water content from the vascular system. The firmness of fruits determines their resistance to mechanical damage^32^. Other studies have shown that smaller fruits had higher firmness^33,34^. The higher firmness of conventional fruits and their puncture is the result of their smaller weight, as in other studies. This contradicts the common opinion that organic fruit is of inferior quality. Despite the similar content of mineral components in the soil on both plantations, supplemented to an optimal level with fertilizers, irrigation or frost protection, organic fruit was much larger. The shrubs that grew on the ecological plantation were also much higher. The influence of the weather on the growth of shrubs can be excluded, because the plantations are about 300 m away from each other. This is confirmed by numerous opinions that highbush blueberry shrubs should be planted in soil with a high level of organic matter and low pH value^7,35^. Optimal growing conditions may also affect the higher resistance of the plants to pathogens, which allows them to be grown without plant protection products^36^.

Fruit firmness is of decisive importance in assessing, among other things, fruit resistance to mechanical damage. It is commonly assumed that such a phenomenon is less intensive in fruits that are well supplied with calcium^37^. Calcium is mainly transported to the leaves, and even a high Ca content in the soil does not guarantee that the fruit firmness will be high^37–39^. In this study, we observed a decrease in fruit firmness after storage. Larger changes in both the firmness and the puncture resistance of the fruit were observed in fruits from organic farming. The mean firmness change after storage was 13.9% and was similar to the value of 15% reported by Chiabrando and Giacalone^40^. Fruit weight loss in ’Brigitta Blue’ was relatively low, from 1.3 to 2%, lower than the 3–5% previously observed after a 45-day storage period at 0 °C. In comparison, weight loss was as high as 15% after 9 weeks of storage under traditional storage conditions^41^. Independent of the cultivation method, the fruits showed similar weight loss during storage. Weight loss in blueberries is mainly due to water loss caused by transpiration and respiration processes, which depend on the gradient of water vapor pressure between the fruits and the surrounding air^40^. In addition to firmness, total acidity, soluble sugar content, and health-promoting elements, consumers highly rate colour of fresh blueberry. The colour of berries is caused by the presence of various bioactive compounds. Orange, red, purple and blue colours are related to the presence of pigments such as anthocyanins, carotenoids and betalains^42^. Changes in the colour of the fruit indicate ripening. A change in the L* parameter, indicating blueberry fruit darkening, was also observed by Chiabrando and Giacalone^40^. Fruits of the Brigitta blue cultivar were also darker after storage, as indicated by a decrease in the L* colour parameter. Organic fruits at the time of harvest were darker than conventional fruits despite being harvested at the same time. It was also found that the change in the colour parameter of organic fruits was greater than that in conventional fruits. The nature of these changes was similar to that in the fruits of the Sunrice cultivar^6^. Ścibisz et al.^43^ demonstrated a significant correlation between anthocyanin content and L* value in experiments with highbush blueberry fruit. However, the research showed that the fruit, despite having similar colours, had a varied contents of anthocyanins. Those harvested from conventional plantations had a much higher content of polyphenolic compounds, including anthocyanins.

### Polyphenol compounds and health promoting capacities

Due to the high content of polyphenolic compounds and high antioxidant activity, blueberries are considered to be functional foods^44,45^. Polyphenols, especially anthocyanins, are mainly found in the skin of blueberries^46^. Smaller fruits have a higher polyphenol content. This is due to the larger skin area of small fruit compared to that in the same amount of large fruit by weight, e.g., 100 g^5^. Moreover, blueberries are characterized by low levels of ingredients that are considered to be toxic. Nitrates are not a major health risk to consumers, but nitrites are produced during the partial reduction of NO3. This process may intensify during transport or storage in conditions with low oxygen content. The presence of these compounds results from fertilization but also from the natural nitrogen cycle^47^. Additionally, cultivation on peat soils, which are rich in organic matter, leads to higher accumulation of nitrates in plants. In sandy soils, nitrate ions are easily leached out. This may explain why the content of harmful compounds in the organic fruit was higher than that in the conventionally grown fruit^48^.

However, the contents of harmful nitrates (max. 48.9) and nitrites (max. 0.24 mg/1000 g) in the tested fruits were low. In accordance with applicable regulations, these fruits can be considered safe for the consumer. Nitrate content limits are set only for green leafy vegetables in EU legislation. Fresh lettuce may contain up to 5000 mg/1000 g nitrate, and processed foods for feeding infants and young children should not exceed 200 mg/1000 g nitrate^49^. In contrast, nitrite levels should not exceed 0.07 mg per kg body weight per day.

In previous studies, the antioxidant activity values ​​measured using ABTS·+ ranged from 811 to 3829 μmol/100 g^50,51^. Differences in antioxidant activity result from the differences among cultivars and among production locations. The levels of antioxidant activity determined by the DPPH and FRAP methods in Bluecrop fruit were 1244 and 700 µmol/100 g, respectively^51^. In both fresh and stored fruits in this study, the activity determined by DPPH was at a higher level than that found in a previous study^51^ (15.69–20.44 µmol/g), and the activity determined by FRAP was at a similar level (7.61–9.35 µmol/g). However, it was found that conventional fruit had a higher polyphenol content and a higher FRAP value than organic fruit. A linear relationship was observed between the total phenolics and FRAP values for blueberries^52^. A similar relationship was also observed in ‘Brigitta Blue’ in this study.

The content of polymeric procyanidins is strongly correlated with the inhibitory activity towards *α*-amylase and *α*-glucosidase^53,54^. Proanthocyanidin-rich blueberry cultivar extracts had the lowest IC_50_ value (25.0 mg/mL), suggesting a high *α*-glucosidase enzyme inhibitory potential^55^. The tested fruit, both fresh and after storage, regardless of the method of cultivation, had a higher ability to inhibit *α*-glucosidase enzymes (IC50 15.07–19.86 mg/mL) than that in a previous study. The high content of polyphenols in the fruit could have an impact on the strong inhibition of *α*-amylase and *α*-glucosidase. The tested fruits were rich in anthocyanins, which inhibit *α*-glucosidase activity and can reduce blood glucose levels after starch-rich meals. Polyphenols are resynthesized products that protect against ultraviolet radiation and pathogens^56^. Anthocyanin compounds are mainly concentrated in the skin of berries^46^. Approximately half as many polyphenols and up to ten times fewer anthocyanins were determined in 'Bluecrop' than in this study^51^. The tests were carried out using a different method, but the very small amounts of anthocyanins call into question the effectiveness of this method for determining these compounds. According to the literature, the anthocyanin content in different blueberry cultivars calculated as equivalents is between 22 and 497 mg/100 g^57,58^. In contrast, in blueberry fruit, the amount of anthocyanins at full maturity determined by HPLC ranged from 245 to 684 mg/100 g^59^.

Flavonoids and phenolic metabolites are main anti-stress phytochemicals that not only enhance nutritional quality of the fruit but also increase its storage life. These phytochemicals protect the plant tissue against damages, especially when the plant is grown in the absence of synthetic pesticides and fungicides. The presence of polyphenolic compounds is an effect of the plants' reaction to stress, and their lower level indicates that the shrubs grew in better conditions than those grown on a conventional plantation. Organically cultivated pears and peaches had higher polyphenolic compound contents than with the conventional system^60^. While, organically cultivation pears and peaches had higher polyphenolic compound contents than with the conventional system. Thus, depending on the species tested are differently affected by the polyphenols content by the cultivation system. Once again, this confirms the opinion that in the cultivation of highbush blueberries, the conditions of cultivation—the appropriate position and soil—have a decisive influence on the quality of the fruit. In this study, a decrease in the content of polyphenols, especially anthocyanins, was observed after storage.

However, after a 45-day fruit storage period, Chiabrando and Giacalone^40^ found a considerable increase in anthocyanin content compared with that in fresh fruit; however, the highest amounts of these compounds were determined after 15 days of storage, and the amounts subsequently decreased.

According to Reque et al.^61^, storing blueberries for six months at − 18 °C resulted in an average anthocyanin degradation of 59%. On the one hand, the anthocyanins contained in plants protect plants from infections because the anthocyanins show antifungal activity^62^. However, some microorganisms are able to biodegrade anthocyanins^63,64^. Microorganisms adapt quickly to the given environmental conditions, and their enzymatic potential, genetic variability and reproductive rate make them capable of neutralizing or even biodegrading many compounds that are toxic to living organisms and using them to obtain macroelements and/or energy^65,66^. Given this information, it seems clear that the microbiological decomposition of polyphenols, tannins and even the cuticle of the epidermis is possible^30^.

Connor et al.^67^ concluded that fruit gathered prior to full ripening can be stored for seven weeks without losses of antioxidants such as flavonols and anthocyanins. However, the polyphenol content of blueberry fruit depends on the phase of ripeness. In some cultivars, the content of polyphenols increases during ripening, while in others, it decreases. A decrease in the content of polyphenols can also occur during storage^68,69^.

## Conclusion

Optimal environmental and soil conditions enabled the organic production of blueberry bushes under certified ecological standards without the use of pesticides and fertilizers. The berries from the organic plantation were larger than the conventionally grown berries by approximately 80% but contained fewer bioactive compounds, namely, polyphenols and *L-*ascorbic acid. The organic growth conditions also resulted in lower antioxidant activity and less α-amylase and α-glucosidase inhibition.

The storage of highbush blueberry fruits in CA cold storage influenced the decrease in polyphenolic compound content; however, it had no significant influence on the decrease in antioxidant activity or on the effectiveness of *α*-amylase and *α*-glucosidase inhibitors.

Regardless of the method of cultivation, after storage, the highbush blueberries became darker, and their firmness decreased.

Fungi that were present on both organic and conventional fruit belonged to the genera *Cladosporium, Penicillium, Alternaria,* and *Aureobasidium*. Their presence on the fruit did not depend on the growth conditions of the crop. Fungi that were found only on the organic fruit belonged to the genera *Bipolaris, Acremonium, Mucor,* and *Fusarium*. These fungi were not found on fruit that were treated with pesticides.

Fresh organic fruit were inhabited by fungi from 8 different genera, while conventionally cultivated fruit were inhabited by fungi from five different genera. Nevertheless, the Shannon–Wiener diversity index parameters and the number of yeasts and moulds were lower for the fresh organic fruit.

The cold storage of fruits for 8 weeks usually decreased the number of yeasts and moulds and changed the make-up of the dominant genera (the dominant genus was *Aureobasidium*). *Fusarium* activity was not eliminated by fruit storage, and zearalenone, which had not been previously detected in fresh fruits, was identified in the fruit samples. In contrast to the conventionally cultivated fruits, the organic fruits were inhabited by fungi of the genus *Fusarium* and contained deoxynivalenol, a mycotoxin.

## Materials and methods

### Cultural conditions

The fruits were harvested from ‘Brigitta Blue’ grown on two farms specialized in the cultivation of highbush blueberry, located in the 20 km to the east of Szczecin^6^. The organic cultivation covered 40 ha of land near a peat mine surrounded by pine forests. Blueberry bushes were planted at a spacing of 1.2–2.3 m in 2003. The bushes grew in high peat (Baltic-type) with acidic pH 3.4–3.9, 55.3% organic matter, 5.8% organic carbon, and electrical conductivity 0.27 mS/cm. Every year in autumn and spring, paraffin oil was sprayed. Every year, KALISOP^®^ (50 kg/ha K_2_O) and Patentkali^®^ (50 kg/ha K_2_O and 20 kg MgO) were applied. The conventional orchard (60 ha) was located on the edge of a forest, approximately 300 m from the organic plantation. The soil is a mineral soil with the particle size of light sand. The bushes grew in mineral soil—loamy sand, pH 4.0–4.2, 2.21% organic matter, 0.56% organic carbon, and EC 0.29 mS/m. Mineral fertilizers were applied annually by means of fertigation, in accordance with the recommendations for highbush blueberry. Ammonium sulfate (200 kg/ha) and the potassium fertilizer KALISOP^®^ (100 kg/ha) were applied. The remaining fertilizers were applied based on soil and leaf analyses. Chemical protection was used as recommended for highbush blueberry. In the leafless period, copper oxychloride spray was sprayed twice. From the beginning of flowering, three sprayings with Signum 33 WG and Switch 62.5 WG were applied. The annual official testing showed that the limit values for pesticide residues in conventional fruit were not exceeded (OJ L70. 16.3.2005).

**Figure 2:**
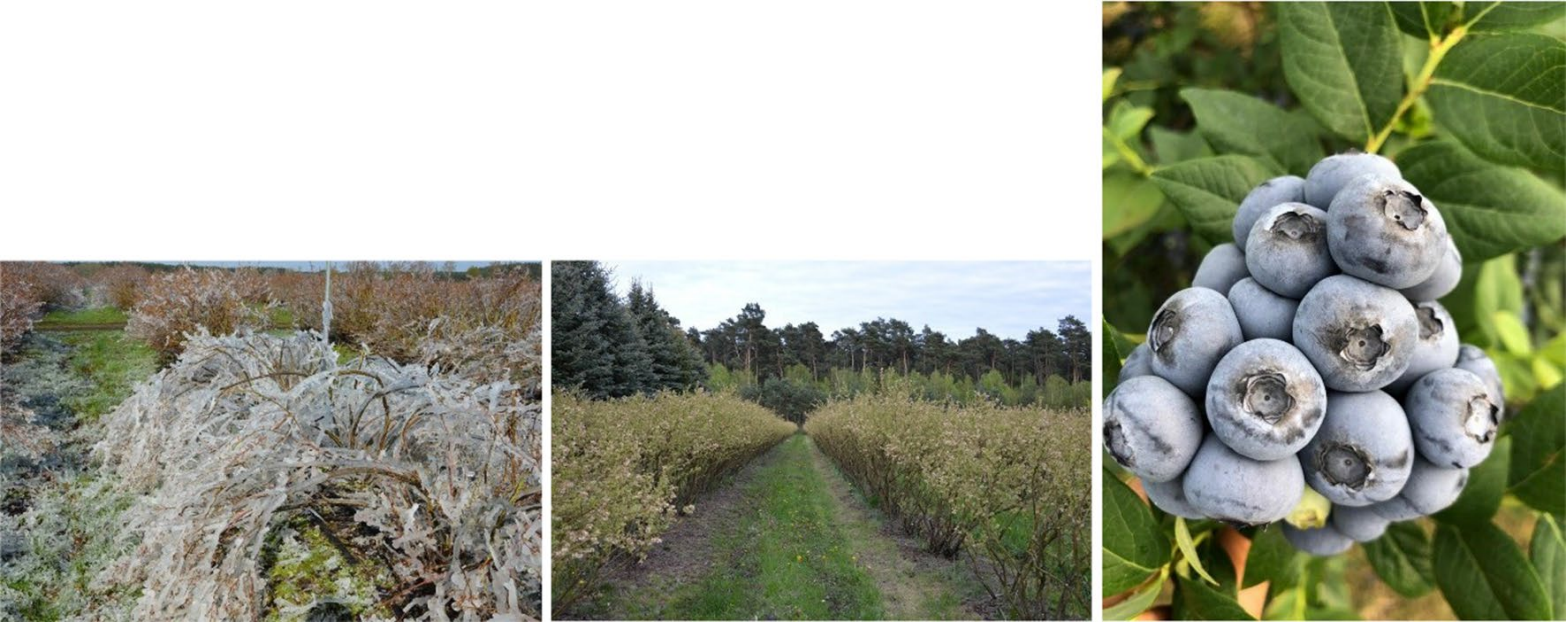
Protection of bushes against frost, organic plantation and highbush blueberry fruit (phot. I. Ochmian).

Every year, during the leafless period, the bushes on both plantations were cut according to the recommendations; approximately 25% of the oldest shoots were cut. In spring, when the temperature dropped below 0 °C, the bushes were sprayed to protect them from spring frosts (Fig. 2). The plantation was irrigated annually using a permanently installed T-Tape drip irrigation line with an emitter performance of 1 L/1 h. The moisture content of the substrates was maintained in the pF 1.8–2.1 range and was determined using contact tensiometers.

### Fruit harvest, sample preparation

The fruit was harvested at full ripeness on the basis of an assessment of the colour of the fruit (it must be fully coloured, without a green mark at the stalk) and the total soluble solids—TSS (13–15%). On each plantation, the fruit were collected in two plots (the plot area is approximately 4 ha) from 25 randomly selected bushes in three repetitions (3 × 25 bushes × 2 plots × 2 cultivation methods). The fruits were harvested by hand four times, each time from the same bushes. The fruits were used to prepare the aggregate samples for analysis. The firmness, skin puncture resistance, and colour of the berry were measured in fresh berries immediately after harvest. The heath prompting compounds: phenolic composition, antioxidant activity, *α*-amylase and *α*-glucosidase inhibitory activity, were determined in berry samples that were kept frozen.

### Fruit and leaf infestation by moulds and yeasts

Branched shoots with leaves, main shoots and fruits were collected at the time of harvesting. The research material was placed in sterile plastic containers. The material was subjected to microbiological analysis (shoots with leaves, main shoots and fruits) immediately after harvesting or after cold storage (blueberry fruits). The analysis of the degree of fruit, shoot and leaf infestation by fungi (yeasts and moulds) was based on the European standard ISO^70^. After the cultivation of spore-forming fungal inoculates, they were subjected to taxonomic evaluation using the traditional method of macroscopic observation of colonies and microscopic observation of spores and filaments^71^. To compare the biodiversity of fungi colonizing fruit from both crops, the Shannon–Wiener index was used. The fungi were identified to the genus level. The Shannon–Wiener index is widely accepted in microbial biocenosis monitoring^72^. Colonies were counted using an automatic colony counter (Alchem PCC04). Analyses were performed on three replicates from each sample.

### Fruit storage in cold CA storage

The shock-cooled berries (temperature drop to 3–4 °C within 2 h after picking) were then stored for 8 weeks in a cold room with a controlled atmosphere (CA: CO_2_-12%; O_2_-1.5%) at a temperature of 1.5 °C ± 0.25 °C. The experiment was performed in five repetitions, each with 1.25 kg of berries, along with fruits intended for sale. There were approximately 10 tons of fruit in the cold chamber. Each sample was in the chamber only with fruit from the test plots. This was to reduce the chance of infection from pathogens from other plots.

### Colour and firmness

Measurements were conducted in CIE SCI L*a*b* system—the full nomenclature is 1976 CIE L*a*b* Space, International Commission on Illumination in Vienna [L* white (100) black (0), a* green (− 100) red (+ 100), b* blue (− 100) yellow (+ 100)], through a 10° observer type and D65 illuminant using a KonicaMinolta CM-700d spectrophotometer. The colour parameters and indices were averaged over 35 measurements^73^. The firmness and puncture resistance of the berry skin were measured with a FirmTech2 apparatus (BioWorks, USA) on 100 randomly selected berries from three replicates. The result was expressed as a gram-force causing fruit surface to bend 1 mm. Measurements were made on the smaller fruit diameter. Punctures were made using a stamp with a diameter of 3 mm^73^.

### Extraction procedure and identification of polyphenol compounds

Three replicates of 1000 g randomly chosen blueberries were kept frozen in polyethylene bags at − 65 °C until analysis, then prepared according to the methodology of Lachowicz et al.^54^. The fruits were extracted with methanol acidified with 2.0% formic acid.

### Inhibitory activities and antioxidant activity

The activity of the fruit extracts was assayed according to the procedure described previously by Podsedek et al.^74^ (*α*-glucosidase) and Nickavar and Уousefian^75^ (*α*-amylase). All samples were assayed in triplicate, and the result was expressed as the IC50. The amount of the inhibitor (expressed as mg of fruit per 1 mL of reaction mixture under assay conditions) required to inhibit 50% of the enzyme activity was defined as the IC_50_ value. For the ABTS·+ (2,2′-azobis(3-etylobenzotiazolino-6-sulfonian) assay, the procedure followed the method of Arnao et al.^76^. The FRAP (ferric-reducing antioxidant power) and DPPH (1,1-diphenyl-2-picrylhydrazyl) assays were conducted according to the method of Brand-Williams et al.^77^. The antioxidant capacity was expressed as mmol Trolox/g dw. The ABTS·+ and FRAP assay measurements were performed with a UV-2401 PC spectrophotometer. The *L-*ascorbic acid and nitrate content were measured with an RQflex 10 requantometer (Merck)^78^.

### The content of mycotoxins in blueberry fruits

The samples were purified on AflaTest immunological affinity columns from Vicam for aflatoxins and with OchraPrep from R-Biopharm Rhóne Ltd. for ochratoxin A, according to the procedure specified by the manufacturer. Patulin, deoxynivalenol, T2, HT2 toxin and zearalenone were analysed by HPLC–MS/MS^79^. The samples were purified on Bond Elut® Mycotoxin columns from Varian. Each sample was subjected to three repetitions.

### Statistical analysis

All statistical analyses were performed with Statistica 12.5 (StatSoft Polska, Cracow, Poland). The data were subjected to one-factor ANOVA. Mean comparisons were performed using Tukey's least significant difference (LSD) test; the significance was set at *p* < 0.05. In addition, the microbial data were analysed using descriptive statistics including percentage, mean and standard deviation (SD) to describe the mould, mycotoxins count and Shannon–Wiener index results.

## Supplementary information

Supplementary Information.
